# A taxonomic review of the genus *Horniolus* Weise from China, with description of a new species (Coleoptera, Coccinellidae)

**DOI:** 10.3897/zookeys.623.10191

**Published:** 2016-10-11

**Authors:** Xiaosheng Chen, Xiufeng Xie, Shunxiang Ren, Xingmin Wang

**Affiliations:** 1 College of Forestry and Landscape Architecture, South China Agricultural University; Guangdong Key Laboratory for Innovative Development and Utilization of Forest Plant Germplasm; 2 Research Center of Agricultural Pest Biocontrol of Guangdong Province, Guangzhou 510642, China; 3 Guangdong Agriculture Industry Business Polytechnic College, Guangzhou 510507, China

**Keywords:** Coccinelloidea, checklist, Hainan Island, Scymnini, taxonomy

## Abstract

Five species of the genus *Horniolus* Weise, 1901 from China are revised, including the description of a new species, *Horniolus hainanensis* Chen & Ren, **sp. n.** *Horniolus sonduongensis* Hoàng, 1979 is reported from China for the first time. A key to the species from China is provided. Nomenclatural history, diagnoses, detailed descriptions, illustrations, and distribution for each species have been provided. A checklist of all known species of this genus is also presented.

## Introduction

The genus *Horniolus* was proposed by Weise (1901), with type species *Horniolus dispar* Weise described from Sri Lanka by monotypy. Bielawski (1961) pointed out that the species described as Scymnus (Pullus) sp. from Sri Lanka by him (Bielawski 1957) was *Horniolus dispar*. Miyatake (1963) provided a detailed description of *Horniolus*, transferred *Scymnus fortunatus* Lewis, 1896 to this genus, and described three additional species from Japan. Subsequently, six more species from China (Hong Kong and Taiwan), India, Malaysia (North Borneo), Thailand and Vietnam were added by him to this genus (Miyatake 1976, 1979). Hoàng (1979) described *Horniolus sonduongensis* from Vietnam. Booth and Pope (1989) transferred *Scymnus guimeti* Mulsant, 1850 to *Horniolus* based on the examination of the type material deposited in the Hope Entomological Collections. Sathe and Bhosale (2001) described *Horniolus mirajensis* from India, but it was synonymized with *Propylea dissecta* (Mulsant, 1850) by Poorani (2004). Recently, Poorani (2015) described *Horniolus sororius* from India. Prior to the present study, only 14 species have been recognized in this genus, occurring from East to South and Southeast Asia, and most species are poorly represented in collections.

*Horniolus* has been placed in the tribe Scymnini, which was included in the subfamily Scymninae by earlier workers such as Sasaji (1971), Pang and Gordon (1986), Fürsch (1996) and Poorani (2002). However, Korschefsky (1931) assigned this genus to the tribe Ortaliini within the subfamily Coccinellinae in his catalogue. Recent phylogenetic study on classification of Coccinellidae using both molecular and morphological data has showed that *Horniolus* Weise and *Rodolia* Mulsant form a clade placed within the tribe Coccidulini in the broadly defined subfamily Coccinellinae (Seago et al. 2011).

Members of *Horniolus* mostly prey on mealybugs, such as *Planococcus lilacinus* Cockerell and *Dysmicoccus brevipes* (Cockerell) (Hemiptera: Pseudococcidae), infesting coffee, tea and pineapple. These beetles also feed on spiraling whitefly, *Aleurodicus dispersus* Russell (Hemiptera: Aleyrodidae), and play an important role in the biological control of this pest (Irulandi et al. 2001, Ramani et al. 2002, He et al. 2013, Poorani 2015).

In the present paper, five species of the genus *Horniolus* from China are revised, with the addition of a new species. All the species are illustrated and described in detail. A key to and distribution map for the species from China are given. A checklist of the species of *Horniolus* around the world is also provided.

## Material and methods

Specimens examined were collected from China and Vietnam, and deposited in the Department of Entomology, South China Agricultural University, Guangzhou, China (SCAU). In addition, the holotypes of *Horniolus novempunctatus* and *Horniolus hisamatsui* were obtained from the collection of the Entomological Laboratory, Ehime University, Matsuyama, Japan (ELEU).

The morphological terms follow Ślipiński (2007) and Ślipiński and Tomaszewska (2010). Measurements were made using a micrometer attached to a SteREO Discovery V20 dissecting stereoscope and are defined as follows:

TW total width, across both elytra at widest part;

TH total height, at highest part of elytra in lateral view;

TL total length, from apical margin of clypeus to apex of elytra;

PL pronotal length, from the middle of anterior margin to the base of pronotum;

PW pronotal width at widest part;

EW elytral width, equal to TW;

EL elytral length, along suture from base to apex including scutellum;

HW head width, at widest part including eyes.

Male and female genitalia were dissected, cleared in a 10% solution of NaOH by boiling for several minutes, and placed on slides for further study. Photographs of the whole beetles and their genitalia were taken according to Chen et al. (2015).

## Taxonomy

### Genus Horniolus

Taxon classificationAnimaliaColeopteraCoccinellidae

Weise, 1901

Horniolus Weise, 1901: 442. Type species: Horniolus dispar Weise, 1901, by monotypy.

#### Diagnosis.

**Figure 1.**
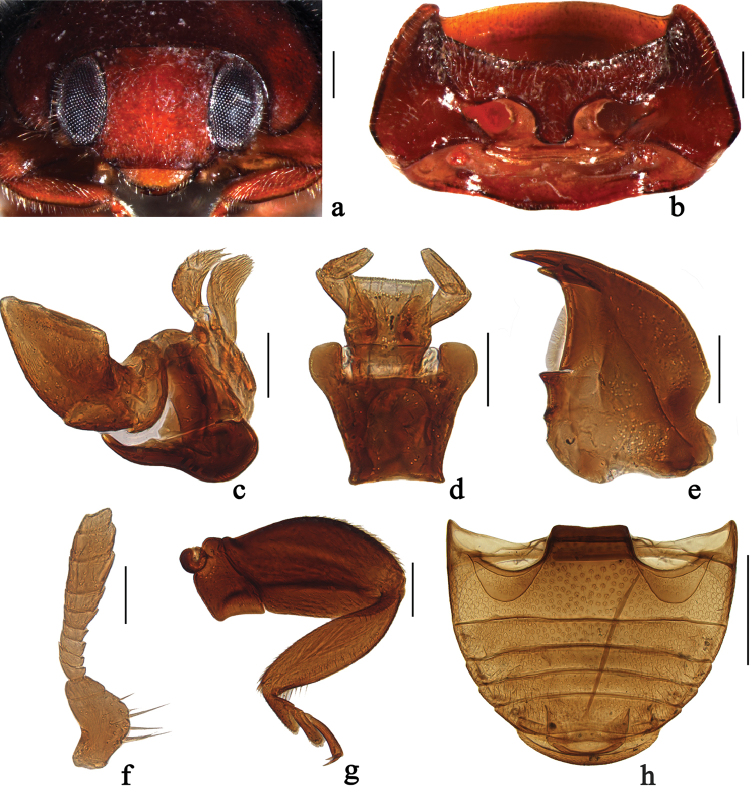
Main characters of the genus *Horniolus* Weise: **a–g** *Horniolus hisamatsui* Miyatake: **a** head **b** prothorax, ventral **c** maxilla **d** labium **e** mandible **f** antenna **g** hind leg **h** *Horniolus sonduongensis* Hoàng: abdomen. Scale bars: 0.2 mm (**a–b, g**), 0.2 mm (**c–f**), 0.5 mm (**h**).

*Horniolus* is similar to *Scymnus* Kugelann, particularly the subgenus Scymnus (Pullus) Mulsant in general appearance. However, it can be easily distinguished from the latter by the following combination of characters: body rounded or elongate oval; antennae composed of 11 antennomeres (Fig. 1f); prosternal process with an inverted Y-shaped carina (Fig. 1b); abdominal postcoxal line complete or apically obliterated and apparently incomplete (Figs 1h, 3d); area enclosed by postcoxal line coarsely punctate; abdomen with six ventrites (Fig. 1h); tarsi with four tarsomeres (Fig. 1g); female genitalia with spermatheca tubular, long and intricately coiled (Fig. 4e).

#### Description.

Body rounded or elongate oval, moderately convex, with dense pubescence, widest around middle of elytra.

Head transverse; frons wide (Fig. 1a). Eyes moderately large, finely faceted, inner ocular margin slightly arcuate. Clypeus truncate anteriorly, slightly expanding laterally, entirely covering antennal insertions. Antennae composed of 11 antennomeres (Fig. 1f); 1^st^ antennomere stout, curved and distinctly rounded on inner side; 2^nd^ firmly united with 1^st^, shorter and narrower than the latter; 3^rd^ obviously trapezoidal, small, outer side nearly two times as long as inner side; 4^th^ to 6^th^ as wide as 3^th^; 7^th^ to 11^th^ forming a fusiform club (Fig. 1f). Labrum exposed, transverse, rounded anteriorly (Fig. 1a). Mandible bifid apically with inner tooth slightly shorter than outer one (Fig. 1e). Terminal maxillary palpomere stout, securiform, weakly broadening apically, apical margin strongly obliquely truncate (Fig. 1c). Labial palps with three palpomeres, terminal palpomere blunt, subcylindrical, shorter than penultimate one (Fig. 1d).

**Figure 2.**
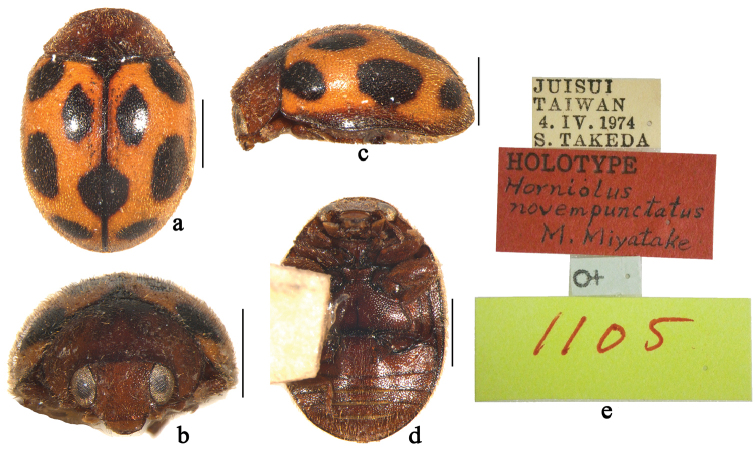
*Horniolus novempunctatus* Miyatake: **a** dorsal view **b** frontal view **c** lateral view **d** ventral view **e** labels of holotype. Scale bars: 1 mm.

**Figure 3.**
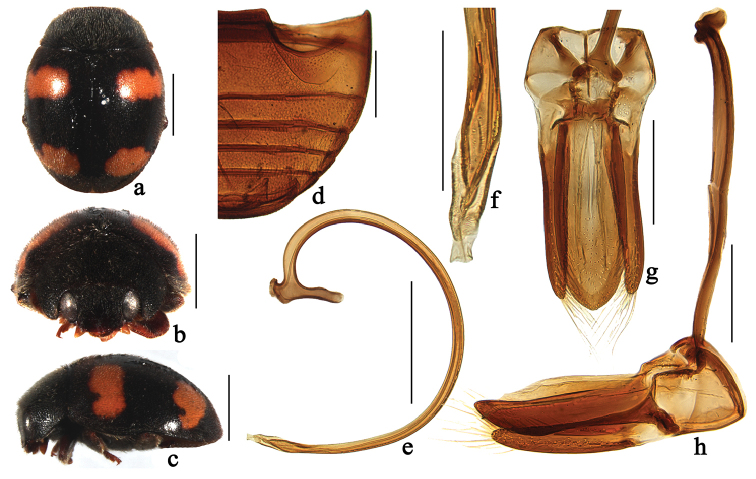
*Horniolus hainanensis* Chen & Ren, sp. n.: **a** dorsal view **b** frontal view **c** lateral view **d** abdomen **e** penis **f** apex of penis **g** tegmen, ventral view **h** tegmen, lateral view. Scale bars: 1 mm (**a–c**), 0.5 mm (**d–e**), 0.2 mm (**f–h**).

Pronotum moderately convex, hind margin wider than anterior one (Fig. 2b). Anterolateral angles of pronotum indistinct, blunt. Pronotal hypomeron broad without delimited foveae (Fig. 1b). Prosternum T-shaped with prosternal process bearing an inverted Y-shaped carina (Fig. 1b), area enclosed by prosternal carina smooth. Scutellum moderately large and triangular (Fig. 2a). Elytra distinctly wider than pronotum at base, surface finely punctate. Elytral epipleuron narrow and nearly horizontal, terminated at level of hind coxae. Abdomen with six ventrites (Fig. 1h). Abdominal postcoxal lines complete or incomplete (Figs 1h, 3d). Legs stout and long (Fig. 1g), not extending beyond external boundary of body; femora of hind leg broad and flattened; tibiae without apical spur; tarsi with four tarsomeres, claws bifid with sharp basal teeth.

#### Distribution.

Bangladesh, China, India, Japan, Malaysia, Nepal, Sri Lanka, Thailand, Vietnam.

#### Key to the species of the genus *Horniolus* from China

**Table:** 

1	Elytra dark brown to black with 4 transverse spots or orange with 6 black spots	**2**
–	Elytra yellow with 9 black spots (Fig. 2a); body length 2.95 mm	***Horniolus novempunctatus* Miyatake**
2	Head and pronotum testaceous; abdominal postcoxal line complete; penis guide shorter than parameres in lateral view	**3**
–	Head and pronotum black (Figs 3a–b); abdominal postcoxal line incomplete (Fig. 3d); penis guide stout, longer than parameres in lateral view (Fig. 3h); body length 2.50–3.20 mm	***Horniolus hainanensis* Chen & Ren, sp. n.**
3	Pronotum entirely reddish brown; apex of penis expanded with membranous appendage	**4**
–	Pronotum dark brown with black marking (Fig. 4a–b); apex of penis convergent apically without membranous appendage, penis guide widest at base in ventral view (Sasaji, 1971); body length 2.83 mm	***Horniolus fortunatus* (Lewis)**
4	Body outline narrower; first pair of elytral spots with hind margin deeply emarginated (Fig. 5a); penis stout and short (Fig. 5i); apex of penis guide strongly curved, forming a hook-shaped in lateral view (Fig. 5l); body length 2.19–2.72 mm	***Horniolus hisamatsui* Miyatake**
–	Body outline broader; first pair of elytral spots with anterior margin deeply emarginated (Fig. 6a); sometimes the orange spots enlarged, each elytron with 2 or 3 black spots (Figs 6d–i); penis slender and long (Fig. 6k); apex of penis guide slightly curved, not forming a hook-shaped projection in lateral view (Fig. 6n); body length 2.76–3.34 mm	***Horniolus sonduongensis* Hoàng**

**Figure 4.**
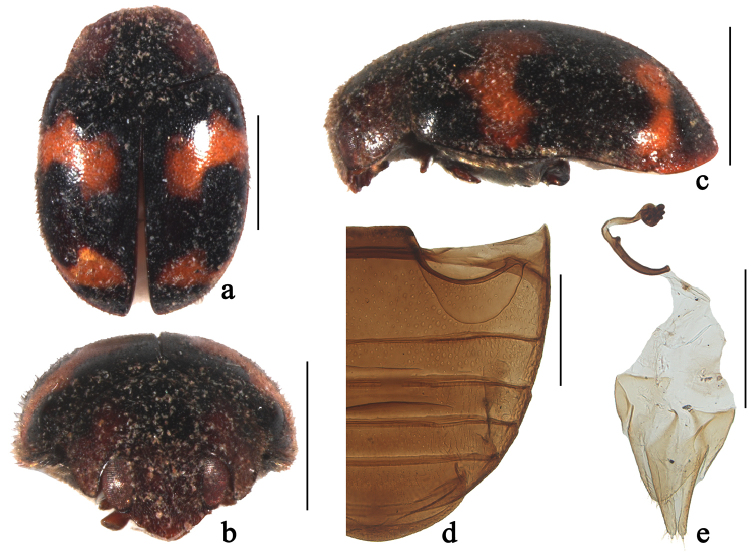
*Horniolus fortunatus* (Lewis): **a** dorsal view **b** frontal view **c** lateral view **d** abdomen **e** female genitalia. Scale bars: 1 mm (**a–c**), 0.5 mm (**d–e**).

**Figure 5.**
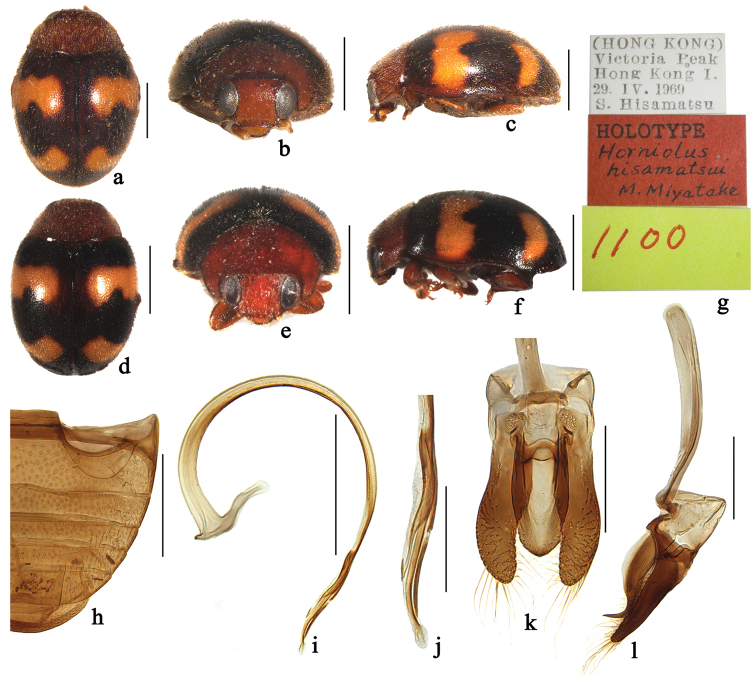
*Horniolus hisamatsui* Miyatake: **a–c** female, holotype **d–f** male **a, d** dorsal view **b, e** frontal view **c, f** lateral view **g** labels of holotype **h** abdomen **i** penis **j** apex of penis **k** tegmen, ventral view **l** tegmen, lateral view. Scale bars: 1 mm (**a–f**), 0.5 mm (**h–i**), 0.2 mm (**j–l**).

**Figure 6.**
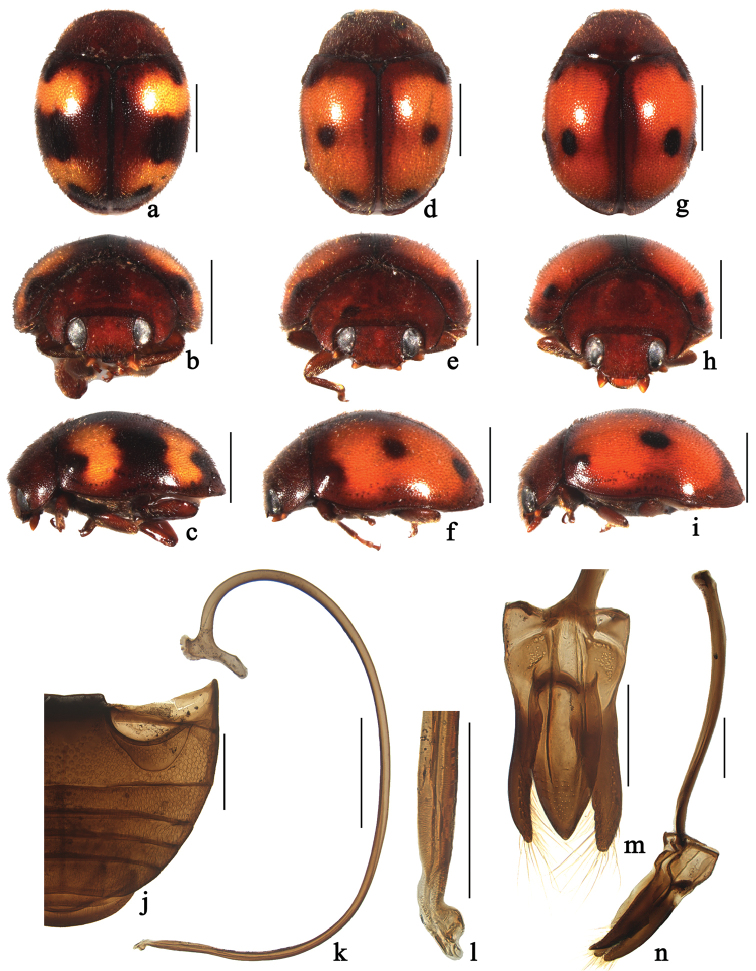
*Horniolus sonduongensis* Hoàng: **a, d, g** dorsal view **b, e, h** frontal view **c, f, i** lateral view **j** abdomen **k** penis **l** apex of penis **m** tegmen, ventral view **n** tegmen, lateral view. Scale bars: 1 mm (**a–i**), 0.5 mm (**j–k**), 0.2 mm (**l–n**).

**Figure 7.**
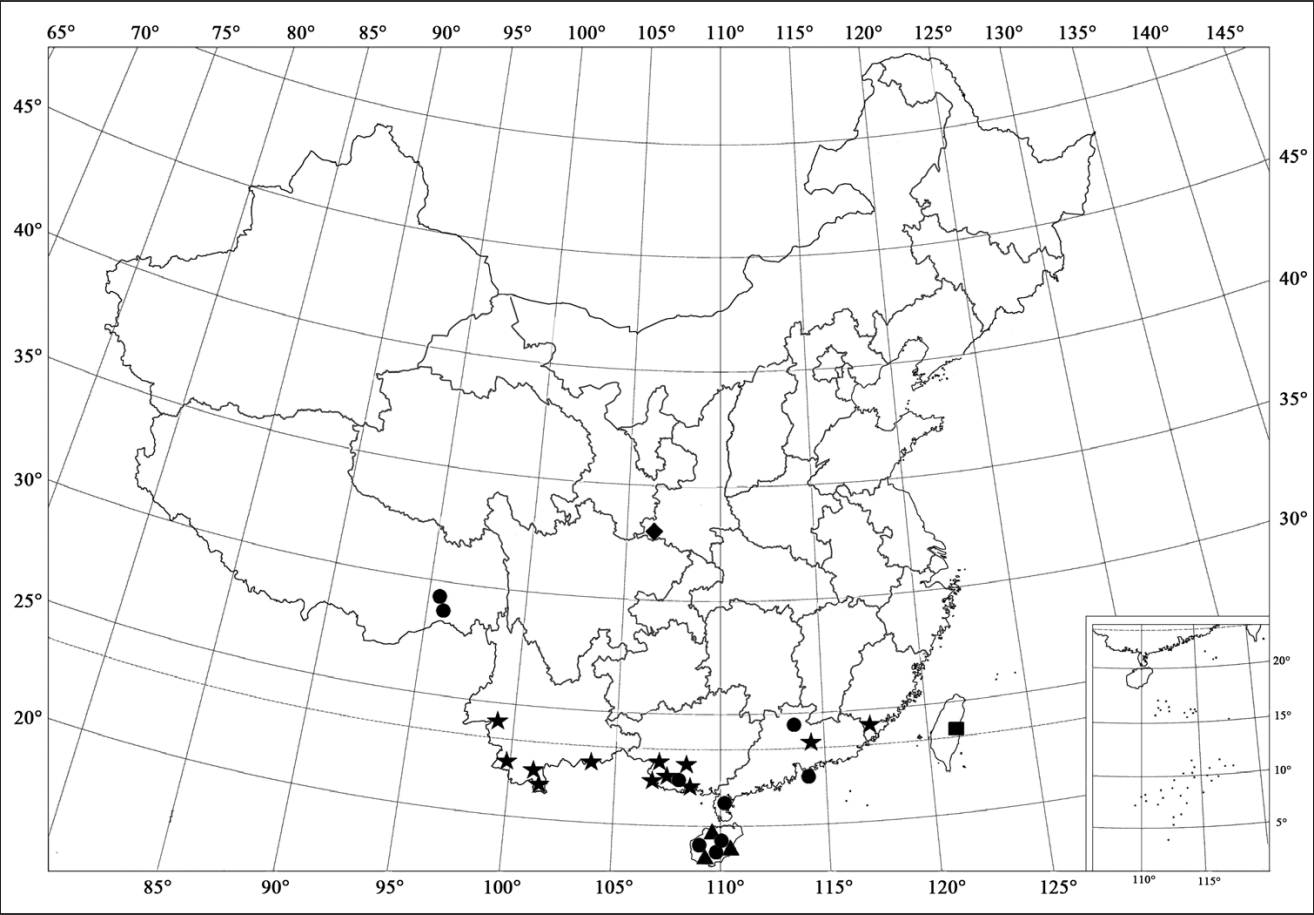
Distribution map. *Horniolus novempunctatus* Miyatake (■); *Horniolus hainanensis* Chen & Ren, sp. n. (▲); *Horniolus fortunatus* (Lewis) (◆); *Horniolus hisamatsui* Miyatake (●); *Horniolus sonduongensis* Hoàng (★).

### Species descriptions and redescriptions

#### Horniolus novempunctatus

Taxon classificationAnimaliaColeopteraCoccinellidae

Miyatake, 1979

Figs 2 , 7

Horniolus novempunctatus Miyatake, 1979: 105; Pang et al. 2004: 89; Kovár 2007: 579.

##### Diagnosis.

This distinctive species can easily be separated from the other species of *Horniolus* by its peculiar colour pattern on elytra.

##### Description.

TL: 2.95 mm, TW: 2.00 mm, TH: 1.52 mm, TL/TW: 1.47, PL/PW: 0.51, EL/EW: 1.12, HW/PW: 0.63, PW/EW: 0.86.

Body elongate oval, moderately convex, dorsum covered with white pubescence (Fig. 2a–c). Head, antennae and mouthparts brown. Pronotum brown to dark brown. Scutellum black. Elytra yellow with nine black spots (Fig. 2a). Underside chestnut (Fig. 2d).

Head with fine frontal punctures, as large as eye facets, 0.5–1.0 diameter apart. Eyes finely faceted, interocular distance 0.56 times head width. Pronotal punctures slightly larger than those on frons, 1.0–2.0 diameters apart. Surface of elytra with punctures larger than those on pronotum, separated by 2.0–3.0 diameters. Prosternal carinae Y-shaped with stem approximately ⅓ as long as arm, arms broadly separated. Abdominal postcoxal lines strongly recurved and complete laterally (Fig. 2d), reaching 4/5 length of abdominal ventrite 1, area enclosed by lines coarsely punctate, narrowly smooth along line. Abdominal ventrite 5 in female with apex rounded.

Male unknown.

##### Type material.

**Holotype**: female, Juisui, Taiwan, [23°31.23'N, 121°24.67'E, ca 300 m], 4. IV. 1974, Takeda S. leg (ELEU, Fig. 2e).

##### Distribution.

China (Taiwan).

#### Horniolus hainanensis

Taxon classificationAnimaliaColeopteraCoccinellidae

Chen & Ren sp. n.

http://zoobank.org/7E71E24F-B0A8-4C5D-BCBD-F337024BAB0C

Figs 3 , 7

##### Diagnosis.

This species is similar to *Horniolus amamensis* Miyatake and *Horniolus kyushuensis* Miyatake in general appearance, but can be separated from the latter by having incomplete abdominal postcoxal lines (Fig. 3d) and broader body outline (Fig. 3a). The stout penis guide (Fig. 3g–h) and the peculiar apex of penis (Fig. 3f) are also diagnostic.

##### Description.

TL: 2.50–3.20 mm, TW: 1.80–2.41 mm, TH: 1.22–1.53 mm, TL/TW: 1.32–1.39, PL/PW: 0.51–0.53, EL/EW: 1.01–1.02, HW/PW: 0.60–0.62, PW/EW: 0.76–0.78.

Body rounded, moderately convex, dorsum covered with white pubescence (Fig. 3a–c). Head black (Fig. 3b). Antennae and mouthparts dark brown. Pronotum and scutellum black. Elytra black with four yellowish spots, first pair transverse, almost straight, parallel to elytral base, situated at anterior ⅓ length of elytra; second pair comma-shaped, located in apical ⅓ length, not reaching suture and lateral margins (Figs 3a, 3c). Prothoracic hypomeron and prosternum black. Mesoventrite and metaventrite black. Elytral epipleuron dark brown with inner and outer margins black. Legs black except tarsi brown.

Head with coarse frontal punctures, distinctly larger than eye facets, 0.5 diameter apart. Eyes finely faceted, interocular distance 0.53 times head width. Pronotal punctures smaller than those on frons, 1.0–2.0 diameters apart. Surface of elytra with punctures larger than those on pronotum, separated by 1.0–2.0 diameters. Prosternal carinae Y-shaped with stem ⅓ as long as arm, arms broadly separated. Abdominal postcoxal lines strongly recurved and distinctly incomplete laterally (Fig. 3d), reaching 4/5 length of abdominal ventrite 1, area enclosed by lines coarsely punctate, narrowly smooth along line. Abdominal ventrite five weakly emarginated apically in male.

Male genitalia. Penis stout, evenly curved (Fig. 3e); penis capsule with long inner arm and short outer arm; apex of penis slightly expanded (Fig. 3f). Tegmen stout (Figs 3g–h) with penis guide parallel-sided from base to ¾ length, then tapering gradually to blunt apex in ventral view (Fig. 3g). Parameres tapering toward apex, shorter than penis guide, densely covered with long setae at apices (Fig. 3h).

Female externally similar to male but with abdominal ventrite 5 truncate apically.

##### Type material.

**Holotype**: male, No. 20070320057, **CHINA: Hainan**: Tongza, 18°54.22'N, 109°40.49'E, ca 470 m, VIII. 1995, Peng ZQ leg (SCAU). **Paratypes (10): Hainan**: 1♀ with same data as holotype. 1♂, Wuzhishan National Nature Reserve, 18°47.07'N, 109°31.98'E, ca 650 m, 22. XI. 1991, Peng ZQ leg. 2♀, Wuzhishan National Nature Reserve, 18°47.07'N, 109°31.98'E, ca 650 m, 8. VI. 1994, Tian MY leg. 1♂, Wuzhishan, 18°47.0'N, 109°31.98'E, ca 650 m, VIII.1995, Peng ZQ leg. 1♂1♀, Lianyuan, Nada, Danzhou City, 19°30.77'N, 109°29.77'E, VIII. 1995, Peng ZQ leg. 1♂, Maoyang Town, Qiongzhong County, 18°56.18'N, 109°30.31'E, ca 230 m, IX.1995, Peng ZQ leg. 1♂, Bawangling National Nature Reserve, 19°05.49'N, 109°06.38'E, ca 260 m, 3. IX. 1998, Peng ZQ leg. 1♂, Limushan National Forest Park, 19°14.05'N, 109°48.03'E, ca 160 m, 22.VII.2006, Dong XL leg (SCAU).

##### Distribution.

China (Hainan).

**Etymology.** The specific epithet refers to its type locality, Hainan Island.

#### Horniolus fortunatus

Taxon classificationAnimaliaColeopteraCoccinellidae

(Lewis, 1896)

Figs 4 , 7

Scymnus fortunatus Lewis, 1896: 38; Ohta 1929: 10.
Scymnus (Scymnus) fortunatus : Mader 1955: 939.
Scymnus (Pullus) fortunatus : Kamiya 1961: 308.
Horniolus fortunatus : Miyatake 1963: 8; Kamiya 1966: 71; Sasaji 1971: 118; Wei et al. 1985: 67; Pang et al. 2004: 89; Kovář 2007: 579.

##### Diagnosis.

This species closely resembles *Horniolus dispar* Weise in elytral pattern and male genitalia, but can be distinguished from it by having dark brown pronotum with black marking (Fig. 4a) and the penis guide distinctly shorter than paremeres (Sasaji 1971).

##### Description.

TL: 2.83 mm, TW: 1.91 mm, TH: 1.24 mm, TL/TW: 1.48, PL/PW: 0.53, EL/EW: 1.08, HW/PW: 0.63, PW/EW: 0.75.

Body elongate oval, slightly convex, dorsum covered with white pubescence (Fig. 4a–c). Head, antennae and mouthparts dark brown. Pronotum dark brown with black marking at middle. Scutellum black. Elytra black with four reddish brown, transverse spots, first pair distinctly sinuated, located before middle in anterior half; second pair smaller, constricted medially, located apical ¼ length of elytra. Prothoracic hypomeron dark brown. Prosternum dark brown to black. Mesoventrite, metaventrite and elytral epipleura black. Legs dark brown.

Head with fine frontal punctures, slightly larger than eye facets, 1.0–2.0 diameters apart. Eyes finely faceted, interocular distance 0.54 times head width. Pronotal punctures as large as those on frons, 1.0–2.0 diameters apart. Surface of elytra with punctures larger than those on pronotum, separated by 2.0–3.0 diameters. Prosternal carinae Y-shaped with stem 1/7 as long as arm, arms narrowly separated. Abdominal postcoxal lines strongly recurved and complete laterally (Fig. 4d), reaching 5/6 length of abdominal ventrite 1, area enclosed by lines coarsely and sparsely punctate, broadly smooth along line.

Male genitalia not studied in the present paper. According to the descriptions and illustrations given by Sasaji (1971: 118), the penis is stout and distinctly convergent apically; penis capsule with long inner arm and short outer arm; apex of penis simple without membranous appendage; tegmen stout with penis guide boat-shaped, widest at base, then tapering gradually to pointed apex in ventral view; parameres strongly curved at base, longer than penis guide, sparsely covered with long setae at apices.

Female externally similar to male except for sexual characters. Abdominal ventrite 5 with apex rounded. Coxites triangular, elongated, outer and inner margins almost straight, tapering to blunt apices, each with dense, long terminal setae (Fig. 4e). Spermatheca tubular, long and intricately coiled (Fig. 4e).

##### Material examined.

**CHINA: Shaanxi**: 1♀, Ningqiang County, 32°49.98'N, 106°14.46'E, ca 850 m, 7. VI. 1982, Collecter unknown (SCAU).

##### Distribution.

China (Shaanxi); Japan.

##### Remarks.

This species has variable pronotal colouration (Miyatake 1963). Lewis (1896) described this species in the genus *Scymnus* Kugelann based on only one specimen from Japan. In his original description, Lewis found a Ceylonese species which closely resembled this species. Ohta (1929) listed this species under *Scymnus* (s. str.) without examining any specimens, and this treatment was followed by Mader (1955). Kamiya (1961) assigned this species to the subgenus Scymnus (Pullus) Mulsant due to the complete abdominal postcoxal line. He also indicated the peculiar character of prosternal carinae and described the male genitalia.

#### Horniolus hisamatsui

Taxon classificationAnimaliaColeopteraCoccinellidae

Miyatake, 1976

Figs 5 , 7

Horniolus hisamatsui Miyatake, 1976: 29; Pang and Gordon 1986: 187; Yu and Lau 2001: 152; Canepari 2003: 262; Pang et al. 2004: 89; Kovář 2007: 579; Ren et al. 2009: 58.

##### Diagnosis.

This species is similar to *Horniolus vietnamicus* Miyatake in general appearance and male genitalia, but can be distinguished from it by the first pair of elytral spots with hind margin deeply emarginated at middle (Fig. 5a, d) and penis guide shorter than parameres (Fig. 5l). In *Horniolus vietnamicus*, the first pair of elytral spots has straight hind margins and penis guide is as long as parameres.

##### Description.

TL: 2.19–2.72 mm, TW: 1.51–1.94 mm, TH: 1.02–1.22 mm, TL/TW: 1.40–1.45, PL/PW: 0.52–0.54, EL/EW: 1.04–1.09, HW/PW: 0.64–0.66, PW/EW: 0.74–0.76.

Body elongate oval, slightly convex, dorsum covered with white pubescence (Fig. 5a–f). Head, antennae and mouthparts reddish brown. Pronotum reddish brown. Scutellum dark brown. Elytra black with 4 yellowish brown, transverse spots, first pair of spots with anterior margins sinuated, hind margins deeply emarginated at middle, located behind middle in anterior half; second pair smaller, comma-shaped, located at apical ¼ length of elytra. Prothoracic hypomeron and prosternum reddish brown. Mesoventrite, metaventrite and elytral epipleura reddish brown. Legs brown.

Head with fine frontal punctures, as large as eye facets, 1.0–2.0 diameter apart. Eyes finely faceted, interocular distance 0.48 times head width. Pronotal punctures larger than those on frons, 1.0–2.0 diameters apart. Surface of elytra with punctures slightly larger than those on pronotum, separated by 2.0–3.0 diameters. Prosternal carinae Y-shaped with stem approximately ⅓ as long as arm, arms broadly separated. Abdominal postcoxal lines strongly recurved and complete laterally (Fig. 5h), reaching ¾ length of abdominal ventrite 1, area enclosed by lines coarsely punctate, broadly smooth along line. Abdominal ventrite 5 in male with apex truncate.

Male genitalia. Penis stout, evenly curved (Fig. 5i); penis capsule with long inner arm and indistinct outer arm; apex of penis slightly expanded with membranous appendage (Fig. 5j). Tegmen stout (Fig. 5k–l) with penis guide parallel-sided at basal half, widest at middle in ventral view (Fig. 5k); in lateral view, penis guide robust at basal ⅔ length, then abruptly narrowed in apical ¼^th^ and produced into a sickle-shaped, acutely pointed apex (Fig. 5l). Parameres narrowed toward apex, longer than penis guide, densely covered with long setae at apices and inner side (Fig. 5l).

Female externally similar to male but with abdominal ventrite 5 rounded apically.

##### Type material.

**Holotype**: female, Victoria Peak, Hong Kong, [22°16.55'N, 114°8.73'E], 29. IV. 1969, S. Hisamatsu leg (ELEU, Fig. 5g).

##### Other material examined.

**CHINA: Guangdong**: 1♂, Shimentai National Nature Reserve, Yangqiu Mountains, 24°16.17'N, 113°17.54'E, ca 500 m, 6.X.2004, Wang XM leg. 2♂, South Subtropical Crops Research Institute, Zhanjiang City, 21°09.78'N, 110°16.45'E, ca 10 m, II. 2012, He YB leg. **Hainan**: 1♂, Yajia, Bawangling National Nature Reserve, 19°04.41'N, 109°09.08'E, ca 970 m, VIII.1995, Peng ZQ leg. 1♂, Tongza, 18°54.22'N, 109°40.49'E, ca 470 m, VIII. 1995, Peng ZQ leg. 2♂, Bawangling National Nature Reserve, 19°05.49'N, 109°06.38'E, ca 260m,5. V. 2005, Wang XM leg. 1♂1♀, Baoting County, 18°37.47'N, 109°41.53'E, ca 70 m, 21.VII.1996, Peng ZQ leg. 1♀, Limushan National Forest Park, 19°14.05'N, 109°48.03'E, ca 160 m, 22.VII.2006, Dong XL leg. 1♀, Wuzhishan National Nature Reserve, 18°47.07'N, 109°31.98'E, ca 650m, 22. XI. 1991, Peng ZQ leg. 1♀, Wuzhishan National Nature Reserve, 18°47.07'N, 109°31.97'E, ca 700m, 3. V. 1996, Peng ZQ leg. 1♀, Yinggen Town, Qiongzhong County, 19°02.10'N, 109°49.84'E, ca 200 m, 15. VII. 1999, Peng ZQ leg. **Guangxi**: 1♂, Pinglongshan, Fulong, Shangsi, 21°49.88'N, 107°56.79'E, ca 160m, 29. VII. 2005, Wang XM leg. **Tibet**: 1♀, Motuo County, Linzhi City, 29°19.30'N, 95°18.33'E, ca 760 m, 17. X. 2009, Chen XS leg. 2♂, Beibeng Village, Motuo County, 29°14.31'N, 95°10.58'E, ca 800 m, 9. X. 2011, Huo LZ et al. leg (SCAU).

##### Distribution.

China (Guangdong, Hong Kong, Hainan, Guangxi, Tibet); Nepal.

#### Horniolus sonduongensis

Taxon classificationAnimaliaColeopteraCoccinellidae

Hoàng, 1979

Figs 6 , 7

Horniolus sonduongensis Hoàng, 1979: 12; 1982: 122; Kuznetsov and Ren 1991: 9.

##### Diagnosis.

This species is similar to *Horniolus vietnamicus* Miyatake and *Horniolus bimaculatus* Miyatake in general appearance and can be separated from these species only by the male genitalia.

##### Description.

TL: 2.76–3.34 mm, TW: 2.03–2.45 mm, TH: 1.40–1.63 mm, TL/TW: 1.35–1.36, PL/PW: 0.54–0.58, EL/EW: 1.02–1.04, HW/PW: 0.60–0.63, PW/EW: 0.72–0.75.

Body rounded oval, moderately convex, dorsum covered with white pubescence (Fig. 6a–i). Head, antennae and mouthparts reddish brown. Pronotum reddish brown. Scutellum dark brown to black. Elytra castaneous to black with 4 orange spots, sinuated (Fig. 6a, c), first pair of spots with anterior margins deeply emarginated at middle, located behind middle in anterior half; second pair smaller, hind margin deeply emarginated medially, located in apical ⅓ length of elytra. Underside entirely castaneous.

Head with coarse frontal punctures, slightly larger than eye facets, 0.2–0.5 diameter apart. Eyes densely faceted, interocular distance 0.54 times head width. Pronotal punctures smaller than those on frons, 2.0–3.0 diameters apart. Surface of elytra with punctures much larger than those on pronotum, separated by 1.0–2.0 diameters. Prosternal carinae Y-shaped with stem approximately ⅓ as long as arm, the arms broadly separated. Abdominal postcoxal lines strongly recurved and complete laterally (Fig. 6j), reaching ¾ length of abdominal ventrite 1, area enclosed by lines coarsely punctate, narrowly smooth along line. Abdominal ventrite 5 in male with apex weakly emarginated medially.

Male genitalia. Penis slender and long (Fig. 6k); penis capsule with long inner arm and short outer arm; apex of penis swollen and curved outwardly (Fig. 6l). Tegmen stout (Fig. 6m–n) with parallel-sided at basal half, then tapering gradually to a blunt apex in ventral view (Fig. 6m). Parameres narrow and curved apically, slightly shorter than penis guide, densely covered with long setae at apices (Fig. 6n).

Female externally similar to male but with abdominal ventrite 5 truncate apically.

##### Material examined.

**CHINA: Fujian**: 1♂, Xiangxi Village, Huboliao National Nature Reserve, Nanjing County, 24°31.07'N, 117°17.08'E, ca 240 m, 18. VIII. 2012, Li WJ leg. **Guangdong**: 1♂5♀, Nankunshan National Nature Reserve, Longmen County, Huizhou City, 23°38.90'N, 113°51.58'E, 460 m, 15. VI. 2014, Ren SX leg. **Guangxi**: 1♂, Pinglongshan, Fulong, Shangsi, 21°49.88'N, 107°56.79'E, ca 160m, 29. VII. 2005, Wang XM leg. 2♂1♀, Naqin Town, Fangchenggang City, 21°49.52'N, 108°02.11'E, ca 100 m, 30.VII.2005, Zhang CW and Wang XM leg. 2♀, Nonggang National Nature Reserve, Longzhou County, 22°28.22'N, 106°57.31'E, ca 230 m, 3. VIII. 2005, Qin ZQ and Zhang CW leg. 2♀, Daqing Mountains, Pingxiang, 24°54.76'N, 113°2.83'E, 2. VIII. 2005, Zhang CW and Wang XM leg. 2♀, Longguang, 29-30. VII. 1985, Pang XF leg. **Yunnan**: 1♂, Xiaowei Mountains, Hekou, 22°53.86'N, 103°34.04'E, ca 900m, 23. IV. 2008, Hao JY leg. 1♂, Mengdui, Zhenkang, 23°53.47'N, 98°53.33'E, 1400m, 18. V. 2008, Liang JB leg. 2♀, Jinuo Mountains, Jinghong, Xishuangbanna, 22°02.21'N, 101°00.35'E, ca 900 m, 6. V. 2009, Chen XS leg. 1♀, Jinuo Mountains, Xishuangbanna, 22°02.21'N, 101°00.35'E, ca 900 m, 28. IV. 2008, Liang JB leg. 1♂2♀, Gongxin, Menglian, 22°18.27'N, 99°19.31'E, 1500 m, 8. V. 2008, Hao JY leg. 1♂, No. 213 Highway, Mengla, 21°33.77'N, 101°34.85'E, ca 700 m, 12-13. X. 2006, Wang XM leg. 1♂, Nature Reserve, Menglun, Mengla, 21°55.27'N, 101°16.64'E, ca 550m, 12-13, X. 2006, Wang XM leg. 1♂, Mengla, Xishuangbanna, 21°26.59'N, 101°38.01'E, ca 1160m, 23. VIII. 2005, Wang XM leg. **VIETNAM**: 1♂, Vietnam, Prov. Gialai-Contum, Buonloi, 14°06.73'N, 107°58.30'E, ca 700 m, 28. V. 1985, Zaitzev U leg (SCAU).

##### Distribution.

China (Fujian, Guangdong, Guangxi, Yunnan) new distribution; Vietnam.

##### Remarks.

This species has variable colour pattern on elytra. In the original description of this species, Hoàng (1979) mentioned it was similar to *Horniolus okinawensis* Miyatake and *Horniolus vietnamicus* Miyatake in colour pattern. In the present study, we found some specimens with orange spots enlarged, but anterior, sutural and lateral margins always castaneous, and each elytron with three black, rounded spots, first one situated on the humerus, second one situated on the middle of elytra, third one situated before apex (Fig. 6d–f); the apical spot disappeared occasionally, only with anterior and middle spots present (Fig. 6g–i).

### Checklist of the species of *Horniolus* Weise, 1901

***Horniolus amamensis* Miyatake, 1963**

*Horniolus amamensis* Miyatake, 1963: 10; Kamiya 1966: 71; Sasaji 1971: 120; Kovář 2007: 579.

**Distribution.** Japan.

***Horniolus bimaculatus* Miyatake, 1976**

*Horniolus bimaculatus* Miyatake, 1976: 35.

**Distribution.** Malaysia.

***Horniolus dispar* Weise, 1901**

*Horniolus dispar* Weise, 1901: 443; Korschefsky 1931: 110; Bielawski 1961: 397; Iablokoff-Khnzorian 1976: 376; Döbler 1987: 37; Poorani 2002: 349.

Scymnus (Pullus) sp.: Bielawski 1957: 83.

**Distribution.** Sri Lanka.

***Horniolus fortunatus* (Lewis, 1896)**

*Scymnus fortunatus* Lewis, 1896: 38; Ohta 1929: 10.

Scymnus (Scymnus) fortunatus: Mader 1955: 939.

Scymnus (Pullus) fortunatus: Kamiya 1961: 308.

*Horniolus fortunatus*: Miyatake 1963: 8; Kamiya 1966: 71; Sasaji 1971: 118; Wei et al. 1985: 67; Pang et al. 2004: 89; Kovář 2007: 579.

**Distribution.** China (Shaanxi); Japan.

***Horniolus guimeti* (Mulsant, 1850)**

*Scymnus guimeti* Mulsant, 1850: 979; Weise 1879: 145; Korschefsky 1931: 143; Booth and Pope 1989: 354.

*Horniolus guimeti*: Booth and Pope 1989: 355; Poorani 2002: 349.

**Distribution.** Malaysia, Bangladesh.

**Notes.** Poorani (2002, 2015) mentioned that *Horniolus guimeti* was a doubtful record for India as suggested by Roger Booth, coccinellid expert at the Natural History Museum, London (BMNH). We agreed with Poorani’s opinion and excluded India in its distribution range.

***Horniolus hainanensis* Chen & Ren, sp. n.**

**Distribution.** China (Hainan).

***Horniolus hisamatsui* Miyatake, 1976**

*Horniolus hisamatsui* Miyatake, 1976: 29; Pang and Gordon 1986: 187; Yu and Lau 2001: 152; Canepari 2003: 262; Pang et al. 2004: 89; Kovář 2007: 579; Ren et al. 2009: 58.

**Distribution.** China (Guangdong, Hong Kong, Hainan, Guangxi, Tibet); Nepal.

***Horniolus kyushuensis* Miyatake, 1963**

*Horniolus kyushuensis* Miyatake, 1963: 9; Kamiya 1966: 71; Sasaji 1971: 119; Kovář 2007: 579.

**Distribution.** Japan.

***Horniolus nigripes* Miyatake, 1976**

*Horniolus nigripes* Miyatake, 1976: 33; Poorani 2002: 350.

**Distribution.** India.

***Horniolus novempunctatus* Miyatake, 1979**

*Horniolus novempunctatus* Miyatake, 1979: 105; Pang et al. 2004: 89; Kovár 2007: 579.

**Distribution.** China (Taiwan).

***Horniolus okinawensis* Chûjô & Miyatake, 1963**

*Horniolus okinawensis* Chûjô & Miyatake in Miyatake, 1963: 11; Kamiya 1966: 71, 85; Sasaji 1971: 121; Chûjô and Chûjô 1998: 19; Kovář 2007: 579.

**Distribution.** Japan.

***Horniolus siamensis* Miyatake, 1976**

*Horniolus siamensis* Miyatake, 1976: 31; Chunram and Sasaji 1980: 476.

**Distribution.** Thailand.

***Horniolus sonduongensis* Hoàng, 1979**

*Horniolus sonduongensis* Hoàng, 1979: 12; 1982: 122; Kuznetsov and Ren 1991: 9.

**Distribution.** China (Fujian, Guangdong, Guangxi, Yunnan); Vietnam.

***Horniolus sororius* Poorani, 2015**

*Horniolus sororius* Poorani, 2015: 7.

**Distribution.** India.

***Horniolus vietnamicus* Miyatake, 1976**

*Horniolus vietnamicus* Miyatake, 1976: 36; Hoàng 1982: 121.

**Distribution.** Vietnam.

## Supplementary Material

XML Treatment for Horniolus

XML Treatment for Horniolus novempunctatus

XML Treatment for Horniolus hainanensis

XML Treatment for Horniolus fortunatus

XML Treatment for Horniolus hisamatsui

XML Treatment for Horniolus sonduongensis
